# Long-term clinical course and outcome in patients with primary Sjögren syndrome-associated interstitial lung disease

**DOI:** 10.1038/s41598-021-92024-2

**Published:** 2021-06-18

**Authors:** Yun Jae Kim, Jooae Choe, Ho Jeong Kim, Jin Woo Song

**Affiliations:** 1 University of Ulsan College of Medicine, Seoul, Republic of Korea; 2 Department of Radiology, University of Ulsan College of Medicine, Asan Medical Center, Seoul, Republic of Korea; 3 Department of Pulmonology and Critical Care Medicine, University of Ulsan College of Medicine, Asan Medical Center, 88 Olympic-ro 43-gil, Songpa-gu, Seoul, 05505 Republic of Korea

**Keywords:** Respiratory tract diseases, Risk factors

## Abstract

Interstitial lung disease (ILD) is the most common lung manifestation in patients with Sjögren syndrome (SJS) and is associated with poor outcomes. This study aimed to investigate the long-term clinical course and prognostic factors in patients with SJS-ILD. Clinical data and high-resolution computed tomography (HRCT) images of 62 patients with primary SJS-ILD were retrospectively analyzed (biopsy-proven cases, n = 16). The mean patient age was 59.8 years; 83.9% of the patients were females, and 38.7% showed a usual interstitial pneumonia (UIP) pattern on HRCT. The median follow-up period was 61.5 months. During follow-up, 15 patients (24.2%) died, 7 (11.3%) experienced acute exacerbation (AE), and 27 (43.5%) progressed. The 1-, 3- and 5-year survival rates were 93.5%, 85.8%, and 81.1%, respectively. Age (hazard ratio [HR]: 1.158, *P* = 0.003), C-reactive protein (CRP) level (HR: 1.212, *P* = 0.045), FVC (HR: 0.902, *P* = 0.005), and a UIP pattern on HRCT (HR: 4.580, *P* = 0.029) were significant prognostic factors in multivariable Cox analysis. In conclusion, death, AE, and ILD progression occurred in 25%, 10%, and 50% of the patients with SJS-ILD, respectively. Older age, higher CRP level, lower FVC, and a UIP pattern on HRCT indicated poor prognosis.

## Introduction

Primary Sjögren syndrome (SJS) is a chronic systemic inflammatory disorder characterized by impaired functioning of the lacrimal and salivary glands^1–3^. Extraglandular organ involvement including that of the skin, lung, heart, kidney, and hematopoietic system has also been reported in SJS^4–9^. Lung involvement is one of the most common extraglandular complications with a recently reported prevalence of 16%, and also reported to be associated with higher mortality in patients with SJS^10,11^. Interstitial lung disease (ILD) is one of the most frequent lung complications^7^. However, the long-term clinical course and prognostic factors in patients with SJS-ILD are not well-defined.

The clinical course and outcomes in patients with SJS-ILD have been reported previously^12,13^. Parambil et al. evaluated 18 patients with SJS-ILD (all biopsy-proven cases) and reported that over a median follow-up period of 67 months, 7 (39%) patients died and 5 (28%) showed progression (decline in forced vital capacity [FVC] ≥ 10% or diffusing capacity of carbon monoxide [DL_CO_] ≥ 15% from baseline)^12^. Similarly, F Roca et al. assessed 21 patients with SJS-ILD and showed that over a median follow-up period of 24 months, 3 (14.3%) patients died and among 19 patients excluding 2 who were lost to follow up, 7 (36.8%) patients showed progression^13^. Some other studies have suggested prognostic factors in patients with SJS-ILD^14,15^. Ito et al. assessed 33 patients with primary SJS showing lung involvement and reported that a lower baseline arterial oxygen pressure (PaO_2_) and presence of microscopic honeycombing on surgical lung biopsy were significant prognostic factors for mortality^15^. Enomoto et al. assessed 33 patients with SJS-ILD (all biopsy-proven cases) and reported that a higher arterial dioxide pressure (PaCO_2_), the extent of reticular abnormality on high-resolution computed tomography (HRCT), and the severity of fibroblastic foci on surgical lung biopsy were risk factors for mortality^14^.

However, previous studies were conducted on a small number of patients, and the long-term clinical course and prognostic factors are still not well-defined in patients with SJS-ILD. Therefore, our study aimed to evaluate the long-term clinical course and prognostic factors in a large number of patients with SJS-ILD.

## Methods

### Study population

Between January 2000 and December 2016, 62 patients with SJS-ILD (biopsy-proven cases: 16) were diagnosed at Asan Medical Center, Seoul, Republic of Korea, and included in this study. All patients were identified through electronic medical record search, and met the criteria of American College of Rheumatology/European League Against Rheumatism^16^. The presence of ILD was confirmed on HRCT images. The study was approved by the Institutional Review Board of Asan Medical Center (2018-1115), and the requirement for informed consent was waived due to the retrospective nature of this study by the Institutional Review Board of Asan Medical Center. All methods were performed in accordance with the relevant guidelines and regulations.

### Clinical data

Clinical and survival data, and pathologic reports for surgical lung biopsy were retrospectively collected (Y.J.K) from medical records, telephone interviews, and/or the records of the National Health Insurance of Korea. Spirometric parameters, total lung capacity (TLC) by plethysmography, and DL_CO_ were measured according to the American Thoracic Society (ATS)/European Respiratory Society (ERS) recommendations^17–19^, and the results were expressed as percentages of normal predicted values. A six-minute walk test (6MWT) was performed according to the ERS/ATS recommendations^20^. Bronchoalveolar lavage (BAL) was performed according to the ATS guidelines^21^.

Data from follow-up assessments at 3–6-month intervals or from hospitalization events were reviewed to determine the development of complications such as pneumonia, acute exacerbation (AE), pulmonary hypertension, or malignant tumors. AE was defined according to the criteria suggested by Collard et al.^22^, as a worsening of dyspnea within 30 days, with new bilateral lung infiltration and no evidence of infection or other alternative causes of dyspnea (e.g., pulmonary embolism or left heart failure). Pulmonary hypertension was measured by transthoracic echocardiography, and a high probability of pulmonary hypertension was defined as a tricuspid regurgitation Vmax of > 3.4 m/s according to the 2015 European Society of Cardiology/ERS guidelines^23^. ILD progression was determined as an absolute decline of at least 10% in the predicted FVC or at least 15% in the DLco^24^.

### HRCT evaluation

HRCT scans were obtained in accordance with standard protocols at full inspiration without contrast enhancement. HRCT scan images were reviewed by a radiologist (J.C.) and a pulmonologist (J.W.S.) blinded to the clinical and pathologic information. Overall, the HRCT pattern was categorized as usual interstitial pattern (definite UIP), probable UIP, indeterminate for UIP, or an alternative diagnosis, based on the idiopathic pulmonary fibrosis (IPF) diagnostic criteria^3^. Alternative diagnosis pattern on HRCT were further categorized according to the 2013 international multidisciplinary classification of idiopathic interstitial pneumonias^25^. Discrepancies were resolved by a consensus. A UIP pattern was defined by a subpleural and basal predominance of reticular abnormalities, honeycombing with or without traction bronchiectasis, and absence of inconsistent findings with a UIP pattern such as extensive ground-glass opacities, micro-nodules, discrete cysts, or segmental/lobar consolidations.

### Statistical analysis

All values are expressed as mean ± standard deviation for continuous variables and as percentages for categorical variables. The Student’s t-test or Mann–Whitney U test was used for analyzing continuous data, and Pearson’s chi-squared or Fisher’s exact test was used for analyzing categorical data. The linear mixed-effects model was used to estimate the pulmonary function changes over time in each patient. The intercept and slope were both fitted as random effects to address the inter-patient differences at baseline and the different rates of pulmonary function changes during the follow-up. We calculated the slope of FVC or DLco to time from the baseline (in years) using FVC or DLco as the dependent variables, and measurement time from the baseline as the independent variable. We used the slope as the annual rate of change in lung function (the absolute change of % predicted value). Changes in lung function were evaluated using a Student’s t-test or analysis of variance (ANOVA) for intergroup comparisons. Kaplan–Meier survival analysis and the log-rank test were performed to evaluate survival. Survival time was calculated as the number of months from the date of diagnosis until death or time of censoring. All patients were followed up during study period, and patients were censored if they were alive on December 31, 2016. The unadjusted or multivariable Cox proportional hazard model was used to evaluate risk factors for mortality. Variables with a *P* value of < 0.1 in the unadjusted analysis were entered into the multivariable models (backward log-likelihood ratio statistics method). All *P*-values were two-tailed, with statistical significance set at a *P* value of < 0.05. All statistical analyses were performed using IBM SPSS Statistics for Windows, Version 24.0. (IBM Corp., Armonk, NY, USA).

## Results

### Baseline characteristics

The baseline clinical characteristics of the patients are summarized in Table 1. Among a total of 62 patients, the mean age was 59.8 years and 83.9% were females. The median follow-up period was 61.5 months (interquartile range, 24.0–99.5 months), and 15 patients (24.2%) died. Major causes of death were underlying ILD progression (66.7%) and pneumonia (13.3%) (see Supplementary Table S1). Total of 53 patients (85.5%) received steroid and/or immunosuppressants (median treatment duration: 15 months [interquartile range: 5–28 months], the initial dose of steroid [mean, prednisolone equivalent dose]: 31.3 mg) (Supplementary Table S2). Non-survivors were older and had less frequent anti-SSA/Ro positivity lower lung function (FVC, DL_CO_, and TLC), poorer exercise capacity (distance and the lowest oxygen saturation during 6MWT), and lower lymphocyte counts in the BAL fluid than survivors (Table 1). Non-survivors also showed a UIP pattern on HRCT more frequently than survivors. However, there were no differences in the treatment during follow-up between non-survivors and survivors.

**Table 1:** Comparison of baseline characteristics between non-survivors and survivors among patients with SJS-ILD.

Characteristics	Total	Non-survivors	Survivors	*P* value
Patient number	62	15	47	
Age, years	59.8 ± 11.4	66.3 ± 9.9	57.7 ± 11.2	0.010
Female sex	52 (83.9)	13 (86.7)	39 (83.0)	> 0.999
Ever-smokers	14 (22.6)	6 (40)	8 (17)	0.082
BMI, kg/m^2^	23.7 ± 3.1	23.5 ± 3.2	23.7 ± 3.1	0.867
C-reactive protein, mg/dL	1.1 ± 2.5	2.4 ± 4.5	0.6 ± 1.1	0.141
ANA, positive (> 1:40)	47 (75.8)	10 (66.7)	37 (78.7)	0.342
Anti SS-A/Ro, positive	48 (77.4)	7 (46.7)	41 (87.2)	0.001
Anti-SS-B/La, positive	25 (40.3)	3 (20.0)	22 (46.8)	0.065
FVC, % predicted	68.2 ± 14.6	60.1 ± 13.1	70.9 ± 14.2	0.012
DL_CO_, % predicted	58.9 ± 18.8	49.3 ± 16.6	61.8 ± 18.6	0.035
TLC, % predicted	71.4 ± 14.1	62.2 ± 12.8	74.0 ± 13.4	0.009
6MWD, meters	420.0 ± 113.7	329.9 ± 123.3	440.8 ± 102.0	0.007
6MWT the lowest SpO_2_, %	91.9 ± 4.4	89.2 ± 4.0	92.5 ± 4.3	0.042
BAL Neutrophil %	12.5 ± 17.3	23.3 ± 28.8	9.2 ± 11.2	0.288
BAL Lymphocyte %	26.4 ± 15.2	13.8 ± 10.6	29.5 ± 14.7	0.021
HRCT patterns				0.006
UIP	24 (38.7)	11 (73.3)	13 (27.7)	
Probable UIP	26 (41.9)	2 (13.3)	24 (51.1)	
Alternative diagnosis	12 (19.4)	2 (13.3)	10 (21.3)	
Treatment with steroid ± IM^a^	53 (85.5)	12 (80)	41 (87.2)	0.674

Data are presented as mean ± standard deviation or number (%), unless otherwise indicated.

*6MWD* six-minute walk test distance, *6MWT the lowest SpO*_*2*_ lowest oxygen saturation during the six-minute walk test, *ANA* anti-nuclear antibody, *BAL* bronchoalveolar lavage, *BMI* body mass index, *DL*_*CO*_ diffusing capacity of the lung for carbon monoxide, *FVC* forced vital capacity, *ILD* interstitial lung disease, *IM* immunosuppressants, *SJS* Sjögren syndrome, *TLC* total lung capacity.

^a^The immunosuppressants included azathioprine (n = 22), cyclosporine (n = 21), mycophenolate mofetil (n = 27), and cyclophosphamide (n = 8).

### Clinical course

The clinical course of patients with SJS-ILD is summarized in Table 2. Of the 62 patients, AE, pneumonia, pulmonary hypertension, and ILD progression occurred in 7 (11.3%), 9 (14.5%), 4 (6.5%), and 27 (43.5%) patients during follow-up, respectively. Lung cancer and lymphoma were not found in any of the patients. Non-survivors experienced more frequent pneumonia, pulmonary hypertension, and ILD progression than survivors; however, there were no differences in the development of AE between the 2 groups (Table 2). Non-survivors also showed higher decline rates in FVC and DL_CO_ than survivors.

**Table 2:** Comparison of clinical course between non-survivors and survivors among patients with SJS-ILD.

Characteristics	Total	Non-survivors	Survivors	*P* value
Patient number	24	26	12	
Acute exacerbation	7 (11.3)	3 (20)	4 (8.5)	0.345
Pneumonia	9 (14.5)	5 (33.3)	4 (8.5)	0.031
Pulmonary hypertension	4 (6.5)	3 (20)	1 (2.1)	0.041
ILD progression	27 (43.5)	10 (66.7)	17 (36.2)	0.038
**Lung function decline rate**^**a**^				
FVC change, %predicted/year	− 1.18 ± 2.04	− 2.77 ± 2.20	− 0.59 ± 1.70	< 0.001
DL_CO_ change, %predicted/year	− 1.45 ± 1.68	− 2.65 ± 1.92	− 1.07 ± 1.41	0.001
TLC change, %predicted /year	0.11 ± 1.91	− 0.45 ± 2.03	0.29 ± 1.86	0.197

Data are presented as mean ± standard deviation or number (%), unless otherwise indicated.

*DL*_*CO*_ diffusing capacity of the lung for carbon monoxide, *FVC* forced vital capacity, *ILD* interstitial lung disease, *SJS* Sjögren syndrome, *TLC* total lung capacity.

^a^Lung function decline rate was defined as the slope of linear function estimated using a linear mixed-effects model with random intercept and random slope.

### Clinical course according to HRCT pattern

**Figure 1:**
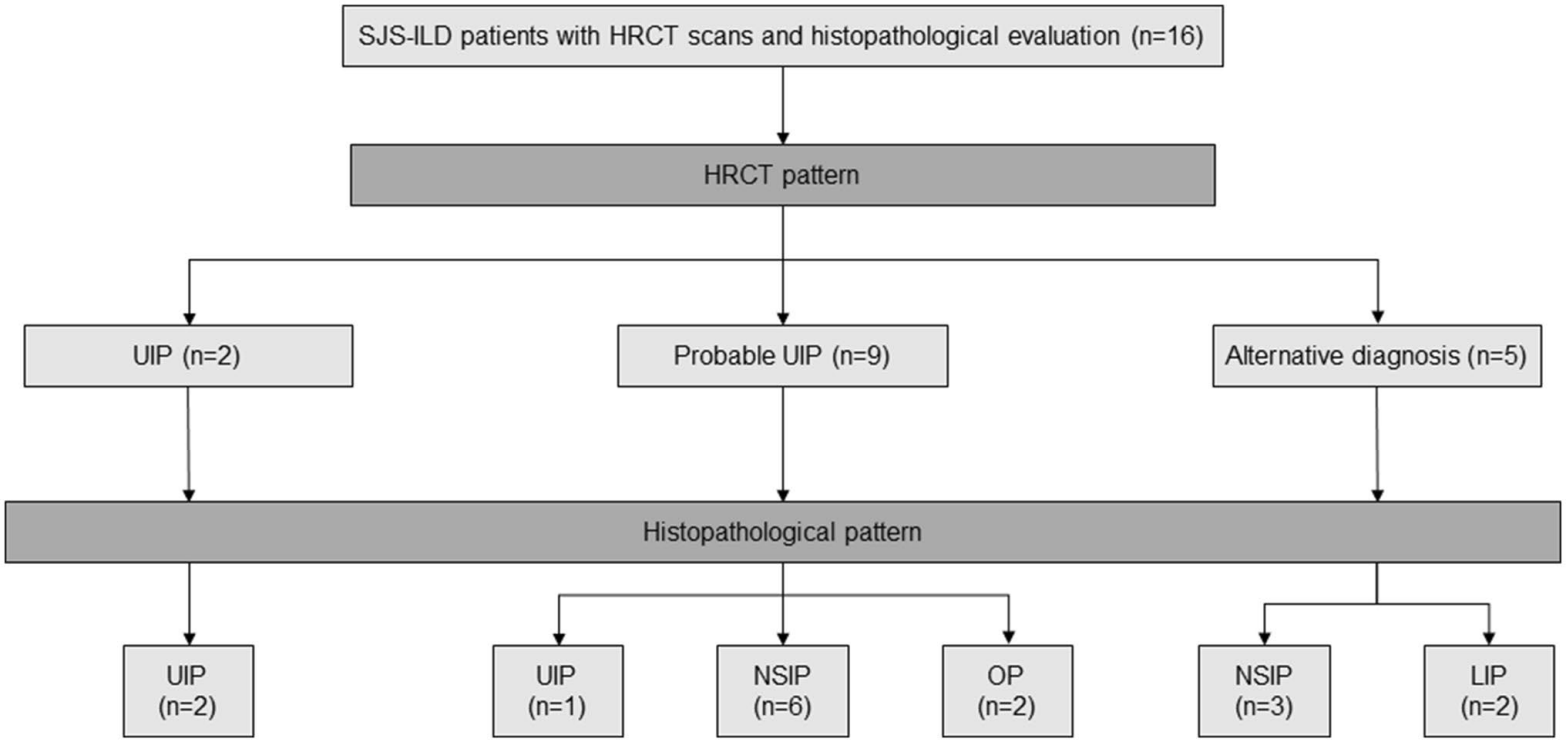
Flowchart of histopathology and HRCT pattern in patients with SJS-ILD. *HRCT* high-resolution computed tomography, *ILD* interstitial lung disease, *LIP* lymphocytic interstitial pneumonia, *NSIP* nonspecific interstitial pneumonia, *OP* organizing pneumonia, *SJS* Sjögren syndrome, *UIP* usual interstitial pneumonia.

Of the 62 patients, 24 (38.7%) were classified as having a UIP pattern on HRCT, 26 (41.9%) as having probable UIP, and 12 (19.4%) as having alternative diagnosis (7 nonspecific interstitial pneumonia [NSIP], 4 lymphocytic interstitial pneumonia [LIP], and 1 organizing pneumonia [OP]). Histopathology was available for 16 patients with SJS-ILD, and radiopathologic correlation are summarized in Fig. 1. All patients (n = 2) with a UIP pattern on HRCT had UIP on biopsy. In patients (n = 9) with a probable UIP pattern on HRCT, NSIP on biopsy was the most common (66.7%), followed by UIP (11.1%) and OP (22.2%). In patients (n = 5) with an alternative diagnosis pattern on HRCT, NSIP on biopsy was also the most common (60.0%), followed by LIP (40%). Baseline characteristics of patients according to the HRCT pattern are summarized in Supplementary Table S3. Patients with a UIP pattern were older and had a shorter walking distance during 6MWT than those in the other groups; however, there was no difference in the prevalence of complications among the 3 groups (Table 3).

**Table 3:** Comparison of clinical course according to HRCT patterns among patients with SJS-ILD.

Characteristics	UIP	Probable UIP	Alternative	*P* value
Patient number	24	26	12	
Acute exacerbation	3 (12.5)	2 (7.7)	2 (16.7)	0.675
Pneumonia	5 (20.8)	2 (7.7)	3 (25.0)	0.092
Pulmonary hypertension	1 (4.2)	2 (7.7)	1 (8.3)	1.000
ILD progression	13 (54.2)	8 (30.8)	6 (50.0)	0.249
**Pulmonary function test**^**a**^				
FVC change, %predicted/year	− 1.81 ± 1.95	− 0.54 ± 2.01	− 0.98 ± 2.06	0.086
DL_CO_ change, %predicted/year	− 2.27 ± 1.70	− 1.00 ± 1.22	− 0.79 ± 1.94	0.007
TLC change, %predicted/year	− 0.46 ± 1.57	0.28 ± 1.54	0.87 ± 2.90	0.122

Data are presented as mean ± standard deviation or number (%), unless otherwise indicated.

*DL*_*CO*_ diffusing capacity of the lung for carbon monoxide, *FVC* forced vital capacity, *ILD* interstitial lung disease, *SJS* Sjögren syndrome, *TLC* total lung capacity, *UIP* usual interstitial pneumonia.

^a^Lung function decline rate was defined as the slope of the linear function estimated using a linear mixed-effects model with random intercept and random slope.

**Figure 2:**
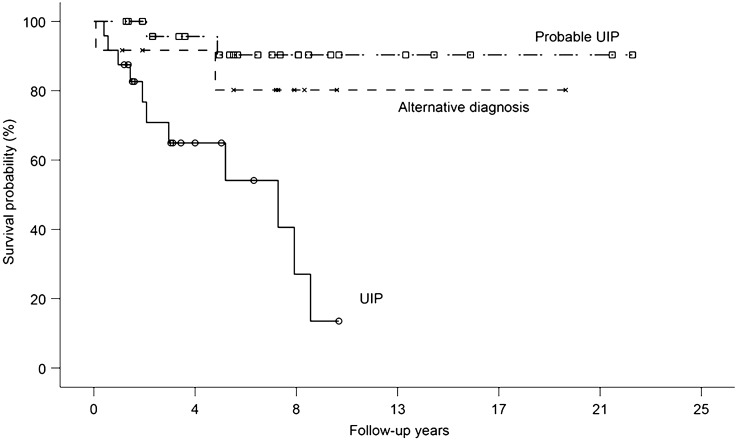
Kaplan–Meier curves of patients with SJS-ILD according to HRCT patterns. Cross marks, open circles, and open squares show censored cases in the alternative diagnosis, UIP, and probable UIP groups, respectively. The prognosis of UIP was significantly different from that of the others (vs. probable UIP; *P* < 0.001, vs. alternative diagnosis; *P* = 0.008, Tukey–Kramer test). The prognosis was not significantly different between probable UIP and alternative diagnosis (*P* = 0.294, Tukey–Kramer test). *HRCT* high-resolution computed tomography, *ILD* interstitial lung disease, *SJS* Sjögren syndrome, *UIP* usual interstitial pneumonia.

The Kaplan–Meier survival curve of patients with SJS-ILD according to the HRCT pattern is shown in Fig. 2. Patients with a UIP pattern on HRCT showed lower survival (median survival, 91 months; 5-year survival, 58.8%) than those with probable UIP (5-year survival, 90.3%, *P* < 0.001) or alternative diagnosis (5-year survival, 80.2%, *P* = 0.008); however, there was no difference in survival between the probable and alternative diagnosis groups (*P* = 0.294). The UIP group also showed a higher decline rate in DL_CO_ and a tendency for a faster decline in FVC and TLC than other groups, but without statistical significance (Table 3).

### Risk factors for mortality

The hazard ratio (HR), 95% confidence interval (CI), and *P* value in the unadjusted analysis for each variable using Cox’s proportional hazard model for the risk of death are shown in Table 4. Statistically significant parameters in the unadjusted analysis were older age, smoking history, higher C-reactive protein (CRP) level, lower FVC, DL_CO_, and TLC, and a UIP pattern on HRCT. In the multivariable analysis, age (HR 1.158; 95% CI 1.050–1.278; *P* = 0.003), CRP level (HR 1.212; 95% CI 1.004–1.462; *P* = 0.045), FVC (HR 0.902; 95% CI 0.839–0.969; *P* = 0.005), and a UIP pattern on HRCT (UIP to non-UIP, HR 4.580; 95% CI 1.167–17.975; *P* = 0.029) were independently associated with survival.

**Table 4:** Prognostic factors for mortality in patients with SJS-ILD assessed using a Cox proportional hazards model.

Characteristics	HR	95% CI	*P* value		HR	95% CI	*P* value
**Unadjusted analysis**				**Multivariable analysis**			
Age, years	1.095	1.036–1.158	0.001	Age, years	1.158	1.050–1.278	0.003
Female sex	1.011	0.226–4.520	0.988				
Ever-smokers	3.206	1.117–9.202	0.030				
CRP	1.294	1.111–1.507	0.001	CRP	1.212	1.004–1.462	0.045
BMI	0.993	0.840–1.173	0.931				
FVC, % predicted	0.958	0.924–0.993	0.021	FVC, % predicted	0.902	0.839–0.969	0.005
DL_CO_^a^, % predicted	0.963	0.932–0.995	0.022				
TLC*, % predicted	0.949	0.912–0.989	0.012				
Treatment with steroid ± IM	0.840	0.236–2.986	0.788				
UIP on HRCT^b^	7.418	2.322–23.693	0.001	UIP on HRCT	4.580	1.167–17.975	0.029

*BMI* body mass index, *CI* confidence interval, *CRP* C-reactive protein, *DL*_*CO*_ diffusing capacity of the lung for carbon monoxide, *FVC* forced vital capacity, *HR* hazard ratio, *HRCT* high-resolution computed tomography, *ILD* interstitial lung disease, *IM* immunosuppressants, *SJS* Sjögren syndrome, *TLC* total lung capacity, *UIP* usual interstitial pneumonia.

^a^Baseline DL_CO_ and TLC were not included in the multivariable analysis due to their close correlation with FVC (vs. DL_CO_, correlation coefficient = 0.710, *P* < 0.001; vs. TLC, correlation coefficient = 0.873, *P* < 0.001).

^b^UIP to non-UIP.

## Discussion

In our study of over 5 years of follow-up, death, AE, and ILD progression occurred in approximately 25%, 10%, and 50% of patients with SJS-ILD, respectively. Older age, higher CRP level, lower FVC, and a UIP pattern on HRCT were significant prognostic factors for mortality in patients with SJS-ILD.

Patients with connective tissue disease-associated interstitial lung disease (CTD-ILD) are known to be at risk of an AE^26,27^. In our study, 6 patients (9.7% of total subjects) experienced an AE. In the study by Suda et al., which retrospectively reviewed 83 CTD-ILD patients with mean observation periods of 6 years, 6 (7.2%) patients developed AE, and 5 of them (83.3%) died despite aggressive anti-inflammatory and immunosuppressive therapy, indicating a poor prognosis of AE in CTD-ILD, which is compatible with that of IPF^27^. In their study, among 17 patients with SJS-ILD, 1 (5.9%) patient developed AE during a mean follow-up period of 6 years^27^. Manfredi et al. assessed 78 patients with CTD-ILD over a mean follow-up period of 23.9 months and reported that 9 (11.5%) patients experienced AE and showed poorer survival than those without AE (overall survival, 31.1% vs. 86.5%; *P* < 0.001)^26^. In their study, among 16 patients with SJS-ILD, 1 (6.3%) patient developed AE^26^.

Our results showed that approximately half of the patients experienced ILD progression during follow-up. Only a few studies have investigated the clinical course of SJS-ILD. Parambil et al. assessed 18 patients with SJS-ILD and reported that 5 (28%) patients showed ILD progression (decline in FVC ≥ 10% or DLco ≥ 15%) over a median follow-up period of 38 months^12^. F. Roca et al. evaluated 19 patients with SJS-ILD and also showed that 7 (36.8%) patients experienced ILD progression (decline in FVC ≥ 10% or DLco ≥ 15%) over a median follow-up period of 24 months^13^. However, our data showed a numerically higher rate of progression than previous reports. This finding might be due to the longer follow-up period in our study than in previous studies, indicating the chronically deteriorating nature of the disease. Moreover, ILD progression may have been overestimated by the calculation method of our study; ILD progression was evaluated based on the lung function value at the time of one follow-up observation. Patients with CTD-ILDs often show some degree of fluctuation in lung function measurements^28^.

Among all participants in this study, a UIP pattern on HRCT was present in 38.7%, which is consistent with the findings of previous reports^13,14^. Roca et al. assessed 21 patients with SJS-ILD and reported that 5 (23.8%) showed a UIP pattern on HRCT^13^. Enomoto et al. evaluated 33 patients with SJS-ILD (all biopsy-proven cases), also demonstrating that 11 (33.3%) patients showed a UIP pattern on HRCT^14^. However, Kamiya et al. evaluated 99 radiologically diagnosed cases of SJS-ILD and reported that when HRCT scans were categorized according to the 2018 Fleischner society guideline, a UIP pattern on HRCT was present in only 9 (9%) cases^29^. Our study also showed that patients with a UIP pattern on HRCT had a poorer clinical course and survival than those without it, and a UIP pattern was an independent prognostic factor in the multivariable analysis. A previous report supports our findings^13^. Roca et al. reported that a UIP pattern on HRCT appeared more frequently in patients with ILD deterioration than in those without (42.9% vs. 16.7%)^13^. However, Kamiya et al. demonstrated that HRCT patterns were not related to poor outcomes in unadjusted (HR 0.41; 95% CI 0.06–3.09; *P* = 0.39) and multivariable (HR 0.40; 95% CI 0.05–3.10; *P* = 0.38) Cox analyses adjusted by age and sex^29^. These different results might be attributable to differences in the study population (prevalence of a UIP pattern, 9% [Kamiya et al.] vs. 38.7% [our study]).

In our study, age, FVC, and a UIP pattern on HRCT were independent prognostic factors in patients with SJS-ILD, and our findings were consistent with the findings of previous reports^14,15,29^. Ito et al. assessed 33 patients with lung involvement in primary SJS and reported that lower PaO_2_ and presence of microscopic honeycombing on surgical lung biopsy were poor prognostic factors for mortality^15^. Enomoto et al. assessed 33 patients with SJS-ILD and also reported that higher PaCO_2_, the extent of reticular abnormalities on HRCT, and the severity of fibroblastic foci on surgical lung biopsy were associated with poorer prognosis^14^. Moreover, Kamiya et al. evaluated 99 patients with SJS-ILD and demonstrated that older age and lower FVC were significantly associated with worse survival^29^. However, our study was the first to show that a UIP pattern on HRCT could be an independent prognostic factor for survival in patients with SJS-ILD.

A probable UIP pattern on HRCT in CTD-ILD is known to have similar prognostic effects as in IPF^30^. Yunt et al., in 158 patients with rheumatoid arthritis (RA)-ILD, reported that there was no difference in survival between patients with definite UIP pattern on HRCT and those with possible UIP (median survival: 8.27 vs. 6.14 years, p = 0.99), but those with definite or possible UIP had worse survival than those with NSIP (p = 0.03)^30^. However, there is also a contradicting finding showing that patients with definite UIP on HRCT have a poorer prognosis than those with probable UIP in CTD-ILD^31^. Jacob et al., in 157 patients with RA-ILD, reported that when CT findings are categorized in 3 groups (definite UIP, probable and inconsistent with UIP), those with a definite UIP pattern (3-year survival: 55%) had similar survival to IPF patients (n = 284, 3-year survival: 42%), but worse than those with a probable UIP (3-year survival: 56%)^31^. In our study, a UIP pattern on HRCT was linked to worse survival compared to probable UIP. This finding might be due to the higher frequency of pathological NSIP pattern in patients with probable UIP pattern on HRCT in SJS-ILD than that in IPF or RA-ILD; in our study, the most common histopathologic pattern was NSIP in patients with radiologic probable UIP pattern (6 out of 9, 66.7%).

This study has some limitations. First, it was a retrospective observational study conducted at a single center. However, the baseline characteristics of the subjects were similar to those reported in other studies^12–15,32^. Second, most of the patients were treated with steroid and/or immunosuppressants, which could have influenced their clinical course and outcomes. However, the treatment was not different between non-survivors and survivors, and thus, was not associated with the prognosis of patients in our study. Finally, the date of diagnosis of patients spans a wide range from 2000 to 2016. However, the survival rates between patients diagnosed before and after 2010 were not different (5-year survival rate: 82.1% and 76.1% respectively, *P* = 0.570). Despite these limitations, our study showed the long-term clinical course and outcomes in a large number of patients with SJS-ILD.

In conclusion, we identified the chronically deteriorating nature of SJS-ILD with around half of the patients experiencing ILD progression over 5 years. Our results suggest that older age, higher CRP, lower FVC and a UIP pattern on HRCT indicate a poorer prognosis in patients with SJS-ILD.

## Supplementary Information

Supplementary Information.

## Data Availability

The datasets generated during and/or analyzed during the current study are available from the corresponding author on reasonable request.
