# Supplementary material for: Long-term clinical course and outcome in patients with primary Sjögren syndrome-associated interstitial lung disease
Source: Sci Rep. 2021 Jun 18;11:12827. doi: 10.1038/s41598-021-92024-2 (PMC8213847; doi:10.1038/s41598-021-92024-2)
Supplement: Supplementary file 1 — Supplementary Information. [file 41598_2021_92024_MOESM1_ESM.docx]

**Long term clinical course and outcome in patients with primary Sjögren syndrome associated interstitial lung disease**

Yun Jae Kim^1^, Jooae Choe^2^, Ho Jeong Kim^3^, Jin Woo Song^3*^

^1^University of Ulsan College of Medicine, Seoul, Republic of Korea.

^2^Department of Radiology, University of Ulsan College of Medicine, Asan Medical Center, Seoul, Republic of Korea.

^3^Department of Pulmonology and Critical Care Medicine, University of Ulsan College of Medicine, Asan Medical Center, Seoul, South Korea.

**Supplementary Table S1.** Causes of death in patients with SJS-ILD

|  | Patients |
| --- | --- |
| Patient number | 15 |
| Underlying ILD progression | 10 (66.7) |
| Pneumonia | 2 (13.3) |
| Tuberculosis | 1 (6.7) |
| Heart failure due to hypertensive heart disease | 1 (6.7) |
| Unknown | 1 (6.7) |

Data are presented as number (%).

SJS-ILD, S[jögren](https://en.wikipedia.org/wiki/Sj%C3%B6gren%27s_syndrome) syndrome associated interstitial lung disease.

**Supplementary Table S2.** Comparison of treatment between non-survivors and survivors among patients with SJS-ILD.

| Characteristics | Total | Non-survivors | Survivors | *P* value |
| --- | --- | --- | --- | --- |
| Treatment with steroid ± IM | 53 | 12 | 41 |  |
| Steroid only | 3 (5.7) | 0 | 3 (7.3) | 1.000 |
| IM only ^a^ | 4 (7.5) | 0 | 4 (9.8) | 0.564 |
| Steroid + IM^b^ | 46 (86.8) | 12 (100) | 34 (82.9) | 0.739 |
| Initial dosage of steroid^c^ | 31.3 ± 12.2 | 32.5 ± 9.4 | 30.1 ± 13 | 0.705 |
| Treatment duration, months^d^ | 15 (5 – 28) | 7.5 (2.3 – 23) | 15 (6 – 31.5) | 0.183 |

IM, immunosuppressants

^a^Cyclosporine (n=4)

^b^The immunosuppressants included azathioprine (n = 22), cyclosporine (n = 17), mycophenolate mofetil (n = 27), and cyclophosphamide (n = 8).

^C^Prednisolone equivalent dose

^d^Median (interquartile range)

**Supplementary Table S3.** Comparisons of baseline characteristics according to HRCT patterns among patients with SJS-ILD

|  | UIP | Probable UIP | Alternative diagnosis | *P* value |
| --- | --- | --- | --- | --- |
| Patient number | 24 | 26 | 12 |  |
| Age, years | 66.3 ± 9.1 | 54.3 ± 11.2 | 58.7 ± 10.2 | <0.001 |
| Female sex | 17 (70.8) | 24 (92.3) | 11 (91.7) | 0.116 |
| Ever-smokers | 9 (37.5) | 3 (11.5) | 2 (16.7) | 0.100 |
| BMI, kg/m^2^ | 23.6 ± 3.9 | 23.8 ± 2.4 | 23.4 ± 3.2 | 0.921 |
| C-reactive protein, mg/dL | 1.6 ± 3.6 | 0.5 ± 0.9 | 1.1 ± 2.0 | 0.353 |
| FVC, %predicted | 68.5 ± 12.7 | 69.7 ± 15.8 | 64.1 ± 16.3 | 0.574 |
| DL_CO_, %predicted | 56.0 ± 15.6 | 64.0 ± 20.5 | 51.3 ± 18.5 | 0.142 |
| TLC, %predicted | 71.5 ± 13.3 | 72.0 ± 13.7 | 69.4 ± 18.0 | 0.901 |
| 6MWD, meters | 352.2 ± 113.0 | 471.3 ± 93.4 | 461.1 ± 76.4 | 0.001 |
| 6MWT the lowest SpO_2_, % | 91.9 ± 4.6 | 92.2 ± 4.6 | 91.3 ± 3.8 | 0.890 |
| BAL Neutrophil % | 16.2 ± 22.9 | 7.3 ± 7.9 | 13.7 ± 8.5 | 0.490 |
| BAL Lymphocyte % | 21.0 ± 14.4 | 32.8 ± 15.4 | 26.3 ± 13.1 | 0.145 |
| Treatment with steroids ± IM^a^ | 19 (79.2) | 23 (88.5) | 11 (91.7) | 0.639 |

Data are presented as mean ± standard deviation or number (%), unless otherwise indicated.

6MWD, six-minute walk test distance; 6MWT the lowest SpO_2_, lowest oxygen saturation during six-minute walk test; BAL, bronchoalveolar lavage; BMI, body mass index; DL_CO_, diffusing capacity of the lung for carbon monoxide; FVC, forced vital capacity; ILD, interstitial lung disease; IM, immunosuppressants; SJS, Sjögren syndrome; TLC, total lung capacity; UIP, usual interstitial pneumonia.

^a^The immunosuppressants included azathioprine (n = 22), cyclosporine (n = 21), mycophenolate mofetil (n = 27), and cyclophosphamide (n = 8).
